# Molecular mechanisms for the destabilization and restabilization of reactivated spatial memory in the Morris water maze

**DOI:** 10.1186/1756-6606-4-9

**Published:** 2011-02-11

**Authors:** Ryang Kim, Ryouichi Moki, Satoshi Kida

**Affiliations:** 1 Department of Bioscience, Faculty of Applied Bioscience, Tokyo University of Agriculture, Tokyo 156-8502, Japan; 2 Core Research for Evolutional Science and Technology, Japan Science and Technology Agency, Saitama 332-0012, Japan

## Abstract

**Background:**

Memory retrieval is not a passive process. Recent studies have shown that reactivated memory is destabilized and then restabilized through gene expression-dependent reconsolidation. Molecular studies on the regulation of memory stability after retrieval have focused almost exclusively on fear memory, especially on the restabilization process of the reactivated fear memory. We previously showed that, similarly with fear memories, reactivated spatial memory undergoes reconsolidation in the Morris water maze. However, the underlying molecular mechanisms by which reactivated spatial memory is destabilized and restabilized remain poorly understood. In this study, we investigated the molecular mechanism that regulates the stability of the reactivated spatial memory.

**Results:**

We first showed that pharmacological inactivation of the N-methyl-D-aspartate glutamate receptor (NMDAR) in the hippocampus or genetic inhibition of cAMP-responsible element binding protein (CREB)-mediated transcription disrupted reactivated spatial memory. Finally, we showed that pharmacological inhibition of cannabinoid receptor 1 (CB1) and L-type voltage gated calcium channels (LVGCCs) in the hippocampus blocked the disruption of the reactivated spatial memory by the inhibition of protein synthesis.

**Conclusions:**

Our findings indicated that the reactivated spatial memory is destabilized through the activation of CB1 and LVGCCs and then restabilized through the activation of NMDAR- and CREB-mediated transcription. We also suggest that the reactivated spatial memory undergoes destabilization and restabilization in the hippocampus, through similar molecular processes as those for reactivated contextual fear memories, which require CB1 and LVGCCs for destabilization and NMDAR and CREB for restabilization.

## Background

To generate long-term memory (LTM), short-term memory is stabilized through a new gene expression-dependent process known as memory consolidation [1-4]. Although it was previously thought that this consolidation occurs once, there is growing evidence indicating that memory stability is changed to either reinforce or alter memory after retrieval [5-11]. In fear conditioning tasks, blocking protein synthesis before or immediately after re-exposure to the conditioning stimulus alone disrupts the subsequent expression of the fear memory [10,12-14]. Such findings suggest that the reactivated fear memory is destabilized and then restabilized through a new gene expression-dependent process, reconsolidation [10,12,13,15].

Abundant studies have focused on and investigated the mechanisms underlying the restabilization of the reactivated fear memory [13,15-21]. For example, we previously demonstrated that the activation of N-methyl-D-aspartate glutamate receptor (NMDAR)- and cAMP-responsible element binding protein (CREB)-mediated transcription is required for the reconsolidation of contextual fear memory [13,14,22]. In contrast, the mechanisms underlying the destabilization after memory reactivation remain poorly understood. Only a few studies, including ours, have shown that the destabilization of reactivated fear memory depends on the activation of NMDAR, proteasome-dependent protein degradation, cannabinoid receptor 1 (CB1), and L-type voltage gated calcium channels (LVGCCs) [23-25]. Indeed, we showed that blockade of the function of CB1 and LVGCCs protects reactivated contextual fear memory from the amnesic effect of blocking protein synthesis [25].

The formation of spatial memory in the Morris water maze depends on the function of the hippocampus in rodents [26]. Abundant studies using molecular genetics and pharmacology have identified key molecules that play essential roles in spatial learning and initial memory formation in the hippocampus [27-31]. On the other hand, we previously showed that the reactivated spatial memory requires protein synthesis-dependent reconsolidation for re-storage in the Morris water maze task [14]. Furthermore, other studies have demonstrated that the reconsolidation of spatial memory depends on hippocampal protein and mRNA synthesis [32-35]. However, the molecular mechanisms underlying the destabilization/restabilization of reactivated spatial memory have not been well characterized.

In this study we investigated the mechanisms for the destabilization and restabilization of reactivated spatial memory using the Morris water maze test. We first showed the mechanism by which reactivated spatial memory is restabilized, where reconsolidation of the reactivated spatial memory is required for the activation of NMDAR in the hippocampus and CREB-mediated transcription. We next showed the mechanisms by which the reactivated spatial memory is destabilized, where destabilization of the reactivated spatial memory is required for the activation of CB1 and LVGCCs in the hippocampus. From these results, we concluded that reactivated spatial and contextual fear memories undergo a similar destabilization/reconsolidation (restabilization) process in the hippocampus.

## Results

### Formation of spatial memory depends on protein synthesis and NMDAR

We previously demonstrated that the reconsolidation of spatial memory requires the synthesis of new proteins [14]. In this previous study, mice were trained to find a hidden platform with 6 trials per day over 2 days [14].

We first tried to confirm that mice can form a spatial memory in this training condition, and tested whether this memory formation requires protein synthesis and the function of NMDAR in the hippocampus as previously shown [31,33,34,36,37].

**Figure 1:**
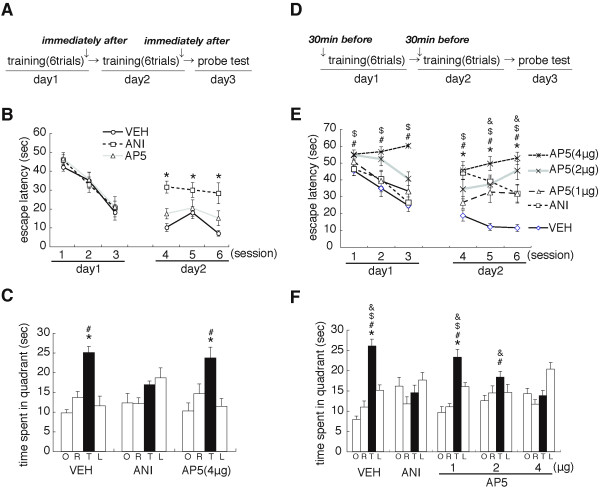
**Roles of protein synthesis and NMDAR in the hippocampus in spatial learning and memory**. (A) Experimental design. (B) Escape latencies during training [VEH, n = 7; ANI, n = 8; AP5 (4 μg/μL), n = 9]. *P < 0.05 compared to the VEH-infused group at each block. Data are indicated in blocks of 2 trials. (C) Probe trial at day 3. *P < 0.05, comparisons of searching scores in the TQ vs. time spent in the other quadrants by *post hoc *Newman-Keuls test after significant one-way ANOVA. ^#^P < 0.05, comparisons of searching scores in the TQ with ANI-infused group. (D) Experimental design. (E) Escape latencies during training [VEH, n = 11; ANI, n = 10; AP5 (1 μg/μL), n = 11; AP5 (2 μg/μL), n = 10; AP5 (4 μg/μL), n = 11] *, ^&, #, $ ^P < 0.05, ANI-, AP5 (1 μg/μL)-, AP5 (2 μg/μL)-, and AP5 (4 μg/μL)-infused groups, respectively, were compared to the VEH-infused group at each block. (F) Probe trial at day 3. *P < 0.05, comparisons of searching scores in the TQ vs. time spent in the other quadrants by *post hoc *Newman-Keuls test after significant one-way ANOVA. ^&, #, $^P < 0.05, comparisons of searching scores in the TQ with ANI-, AP5 (2 μg/μL)- and AP5 (4 μg/μL)-infused groups, respectively. Error bars are SEM. Time spent (s) in target (T), adjacent left (L), adjacent right (R), and opposite (O) quadrants during the probe trial is shown.

Mice were trained on days 1 and 2 as described above and received micro-infusions of the protein synthesis inhibitor anisomycin (ANI) or the vehicle (VEH) into the dorsal hippocampus immediately after the daily training session (Figure 1A). Twenty-four hours after training (day 3), mice were given a probe trial in which the platform was removed from the pool. ANI-infused mice showed worse memory performance during training at day 2, but not at day 1, compared to control mice treated with VEH. A repeated-ANOVA comparing escape latencies revealed a significant effect of drug (VEH vs. ANI) at day 2, but not at day 1, although there was no significant effect of drug versus session (day 1, drug, F_1,13 _= 0.338, P > 0.05; session, F_2,26 _= 19.994, P < 0.05; drug vs. session, F_2,26 _= 0.325, P > 0.05; day 2, drug, F_1,13 _= 32.794, P < 0.05; session, F_2,26 _= 1.629, P > 0.05; group vs. session, F_2,26 _= 1.185, P > 0.05). Consistently, the *post hoc *Bonferroni's test showed that ANI-infused mice displayed significantly longer escape latencies at day 2 than VEH-infused mice (P < 0.05) (Figure 1B). These results indicated that the inhibition of protein synthesis in the hippocampus following training at day 1 impaired the spatial memory. In the probe test at day 3, VEH-infused, but not ANI-infused, mice searched selectively in the target quadrant (TQ) of the pool where the platform was previously located (Figure 1C). A one-way ANOVA revealed a significant effect of group [TQ, opposite (OP), adjacent right (AR), and adjacent left (AL) quadrants] (VEH, F_3,24 _= 16.293, P < 0.05; ANI, F_3,28 _= 2.822, P > 0.05). The *post hoc *Newman-Keuls test showed that the ANI-infused group showed no preference for the target location (P > 0.05), whereas VEH-infused mice displayed significantly longer searching scores in the TQ (time spent in the TQ) than in the other quadrants (P < 0.05). Consistently, ANI-infused mice showed significantly lower searching scores in the TQ than VEH-infused mice (P < 0.05). These results confirmed that ANI-infused mice failed to form a spatial memory. Collectively, our observations confirmed the previous findings that protein synthesis in the hippocampus is required for the formation of spatial memory [33,34].

It is important to note that in our experimental conditions, the infusion of ANI into the dorsal hippocampus inhibited the induction of the immediate early gene c-fos by a systemic administration of the chemical convulsant pentylenetetrazol (PTZ) (Additional file 1, Figure S1), confirming previous observations [25,38].

We next examined the effect of micro-infusions of an NMDAR antagonist on the formation of spatial memory (Figure 1). Mice were trained as described above and received micro-infusions of the NMDAR antagonist AP5 (4 μg/μL) into the dorsal hippocampus immediately after the daily training session (Figure 1A). In contrast to the results for the micro-infusion of ANI, AP5-infused mice showed comparable memory performance during training at days 1 and 2 with VEH-infused mice [day 1, drug (VEH vs. AP5), F_1,14 _= 0.427, P > 0.05; session, F_2,28 _= 23.147, P < 0.05; drug vs. session, F_2,28 _= 0.157, P > 0.05; day 2, F_1,14 _= 2.917, P > 0.05; session, F_2,28 _= 4.416, P < 0.05; drug vs. session, F_2,28 _= 0.587, P > 0.05] (Figure 1B). Consistently, AP5- and VEH-infused mice searched selectively in the TQ in the probe test at day 3 (Figure 1C; VEH, F_3,32 _= 6.870, P < 0.05; AP5, F_2,21 _= 4.552, P < 0.05) and showed comparable searching scores in the TQ (P > 0.05). These observations indicated that the post-training infusion of AP5 into the hippocampus did not affect the formation of spatial memory in our experimental design.

Previous studies suggested that spatial learning and memory depend on the function of hippocampal NMDAR [31]. Therefore, we further examined the effects of a pre-training infusion of drugs (VEH, ANI, or AP5) into the dorsal hippocampus. We performed a similar experiment as in Figure 1A-C, except that mice received micro-infusions of drugs into the dorsal hippocampus at 30 min before the daily training session (Figure 1D). Similarly with the results shown in Figure 1B, the micro-infusion of ANI impaired memory performance during training at day 2, but not at day 1 (day 1, drug, F_1,19 _= 0.563, P > 0.05; session, F_2,38 _= 16.724, P < 0.05; drug vs. session, F_2,38 _= 0.217, P > 0.05; day 2, drug, F_1,19 _= 50.247, P < 0.05; session, F_2,38 _= 5.273, P < 0.05; drug vs. session, F_2,38 _= 0.628, P > 0.05) (Figure 1E). Consistently, in the probe test at day 3, ANI-infused mice showed no preference for the TQ (F_3,36 _= 1.697, P > 0.05), whereas VEH-infused mice searched selectively in the TQ compared to the other quadrants (F_3,40 _= 33.255, P < 0.05) (Figure 1F). ANI-infused mice showed significantly lower searching scores in the TQ than VEH-infused mice (P < 0.05). These observations indicated that pre-treatment with ANI at 30 min before training was sufficient to block the formation of spatial memory.

Interestingly, the pre-training infusion of AP5 into the dorsal hippocampus impaired memory performance during training at both days 1 and 2 (Figure 1E). A repeated ANOVA across drugs [VEH, and low-, middle-, and high-doses of AP5 (1, 2 and 4 μg/μL)] revealed significant effects of drugs and a drugs versus session interaction at day 1 (drug, F_3,39 _= 14.778, P < 0.05; session, F_2,78 _= 9.516, P < 0.05; drug vs. session, F_2,78 _= 2.609, P < 0.05) and a significant effect of drugs at day 2 (drug, F_3,39 _= 25.387, P < 0.05; session, F_2,78 _= 10.041, P > 0.05; drug vs. session, F_2,78 _= 1.750, P > 0.05). Consistently, the middle- and high-, but not low-, dose AP5-infused mice showed significantly longer escape latencies during training at days 1 and 2 than VEH-infused mice (P < 0.05) (Figure 1E). In the probe test at day 3, low-, but not middle- or high-, dose AP5-infused mice searched selectively in the TQ compared to the other quadrants (low, F_3,40 _= 27.181, P < 0.05; middle, F_3,36 _= 2.226, P > 0.05). Although one-way ANOVA detected a significant effect of group in the high-dose AP5-infused mice (F_3,40 _= 7.517, P < 0.05), these mice failed to display significantly higher searching scores in the TQ compared to the other quadrants. Consistently, one-way ANOVA comparing the time spent in the TQ revealed a significant effect of group (F_3,39 _= 13.344, P < 0.05). High-dose AP5-infused mice showed significantly lower searching scores in the TQ compared to all of the other groups (P < 0.05), whereas middle-dose AP5-infused mice showed significantly lower scores than the VEH- and low-dose AP5-infused mice (P > 0.05). These observations indicated that the infusion of AP5 into the dorsal hippocampus impaired spatial learning and memory in a dose-dependent manner.

It is possible that blocking the formation of spatial memory by ANI and AP5 was due to the disruption of the hippocampus function, and not target inhibition of signal transduction such as protein synthesis- and NMDAR-mediated signals. To examine these possibilities, we performed a contextual fear conditioning task, known as the hippocampus-dependent task [39], using mice assessed spatial memory as shown in Figure 1D-F (Additional file 2, Figure S2). Mice were trained with a single footshock (0.4 mA) in the context chamber (training) and freezing was assessed in the same context for 5 min at 24 h after training (test). Twenty-four hours following training, ANI- and AP5-infused mice showed comparable levels of freezing compared to VEH-infused mice (F_4,42 _= 0.683, P > 0.05), suggesting that ANI- and AP5-infused mice formed normal contextual fear memories. These observations suggest that treatment with neither ANI nor AP5 led to significant damage of hippocampal function.

Collectively, these results confirmed previous findings and indicated that spatial learning and memory depends on NMDAR and protein synthesis. It is also suggested that NMDAR and protein synthesis display distinct time windows for spatial learning and memory; NMDAR is required at an earlier stage compared to protein synthesis.

### Reconsolidation of spatial memory depends on protein synthesis and NMDAR

**Figure 2:**
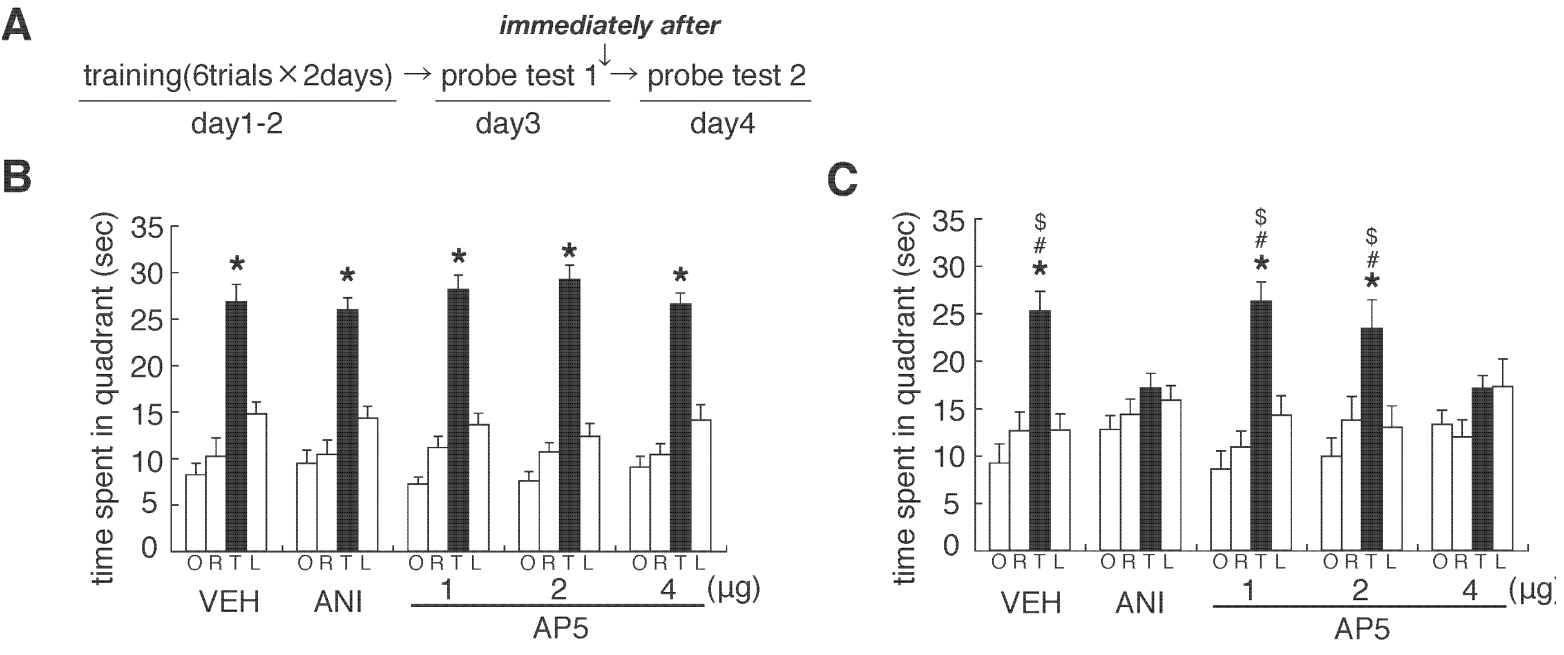
**Roles of protein synthesis and NMDAR in the hippocampus in the reconsolidation of spatial memory**. (A) Experimental design. (B) Probe trial at day 3 (PT 1) [VEH, n = 9; ANI, n = 11; AP5 (1 μg/μL), n = 10; AP5 (2 μg/μL), n = 12; AP5 (4 μg/μL), n = 13]. *P < 0.05, comparisons of searching scores in the TQ vs. time spent in the other quadrants by *post hoc *Newman-Keuls test after significant one-way ANOVA. (C) Probe trial at day 4 (PT 2). *P < 0.05, time spent in the TQ vs. time spent in the other quadrants by *post hoc *Newman-Keuls test after significant one-way ANOVA. ^$, #^P < 0.05, comparisons of searching scores in the TQ with ANI- and AP5 (4 μg/μL)-infused groups, respectively. Error bars are SEM. Time spent (s) in target (T), adjacent left (L), adjacent right (R), and opposite (O) quadrants during the probe trial is shown.

We previously demonstrated that reactivated spatial memory requires protein synthesis-dependent reconsolidation for re-storage [14]. Furthermore, other studies extended this finding and indicated that the reconsolidation of spatial memory depends on protein synthesis in the hippocampus [[33,35], but also see [34]]. To further clarify the mechanisms for the reconsolidation of reactivated spatial memory, we first examined the roles of hippocampal protein synthesis and NMDAR in the reconsolidation of spatial memory (Figure 2).

Mice were trained over 2 days (days 1 and 2) as described above (Figure 1). Twenty-four hours after training (day 3), the first probe trial was performed to reactivate the spatial memory [probe test 1 (PT 1), Re-exposure]. Immediately after PT 1, mice received a micro-infusion of drugs (VEH, ANI, or AP5) into the dorsal hippocampus. Twenty-four hours after PT 1 (day 4), the second probe trial (PT 2, Test) was performed to assess spatial memory. In these experimental protocols, we previously observed that a systemic injection of ANI disrupted reactivated spatial memory [14].

Similarly with the results presented in Figure 1, all groups of mice showed comparable memory performance and a progressive loss of escape latencies during training (days 1 and 2) (data not shown). Twenty-four hours after training on day 2, all groups of mice selectively searched the TQ in PT 1 (VEH, F_3,32 _= 26.244, P < 0.05; ANI, F_3,40 _= 29.418, P < 0.05; AP5 1 μg/μL, F_3,36 _= 55.537, P < 0.05; AP5 2 μg/μL, F_3,44 _= 60.396, P < 0.05; AP5 4 μg/μL, F_3,52 _= 36.892, P < 0.05). Consistently, comparisons of the time spent in the TQ showed that all groups displayed comparable searching scores in the TQ (P > 0.05, Figure 2B). These results indicated that all groups formed and reactivated spatial memory.

Twenty-four hours after PT 1, VEH-infused, but not ANI-infused, mice selectively searched the TQ in PT 2 (VEH, F_3,32 _= 13.314, P < 0.05; ANI, F_3,40 _= 1.524, P > 0.05) (Figure 2C). ANI-infused mice showed a significant decrease in searching scores in the TQ in PT 2 than in PT 1 (F_1,20 _= 18.454, P < 0.05) and significantly lower searching scores than VEH-infused mice in PT 2 (F_1,18 _= 10.435, P < 0.05). These observations indicated that inhibition of protein synthesis in the hippocampus disrupted spatial memory after reactivation, confirming the previous finding that hippocampal protein synthesis is required for the reconsolidation of spatial memory [33,35].

Similarly with the results of ANI, the blockade of NMDAR in the hippocampus also impaired the reactivated spatial memory (Figure 2C). The low-(1 μg/μL) and middle-(2 μg/μL), but not high-(4 μg/μL), dose AP5-infused mice selectively searched the TQ in PT 2 (low, F_3,36 _= 16.937, P < 0.05; middle: F_3,44 _= 5.665, P < 0.05; high, F_3,52 _= 1.970, P > 0.05). Consistently, high-dose AP5-infused mice showed a significant decrease in searching scores in the TQ compared to PT 1 (P < 0.05). Additionally, a one-way ANOVA comparing searching scores in the TQ in PT 2 among VEH- and AP5-infused mice revealed a significant effect of group (F_3,41 _= 3.984, P < 0.05). High-dose AP5-infused mice showed significantly lower searching scores in the TQ than VEH- and low-dose AP5-infused mice (P < 0.05). The middle-dose AP5-infused group showed slightly lower and higher searching scores in the TQ than the low- or high-dose AP5-infused groups, respectively, although these differences were not statistically significant. These observations indicated that the inhibition of NMDAR in the hippocampus impaired reactivated spatial memory in a dose-dependent manner, suggesting that the activation of hippocampal NMDAR is required for the reconsolidation of spatial memory (Figure 2C).

Similarly with Figure 1, we performed a contextual fear conditioning test using mice assessed the reconsolidation of spatial memory as shown in Figure 2 (Additional file 2, Figure S2) and observed that all groups of mice formed normal contextual fear memory (F_4,51 _= 0.423, P > 0.05). These observations suggest that treatment with neither ANI nor AP5 led to significant damage of the function of the hippocampus.

**Figure 3:**
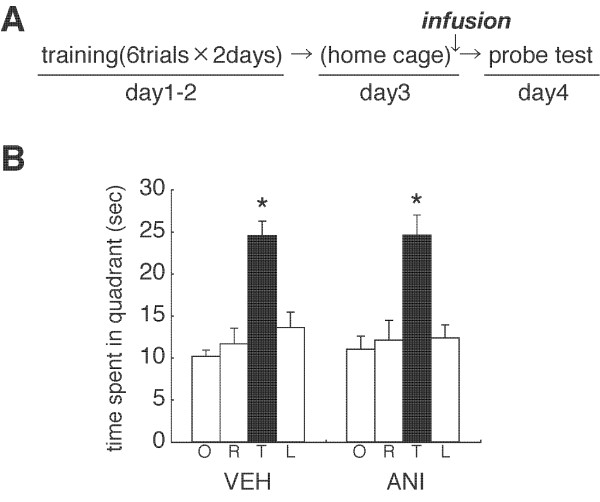
**Effect of no re-exposure on the stability of spatial memory**. (A) Experimental design. (B) Probe trial at day 4 (VEH, n = 9; ANI, n = 9). *P < 0.05, time spent in the TQ vs. time spent in the other quadrants by *post hoc *Newman-Keuls test after significant one-way ANOVA. Error bars are SEM. Time spent (s) in target (T), adjacent left (L), adjacent right (R), and opposite (O) quadrants during the probe trial is shown.

As a control experiment, we examined whether the disruption of spatial memory by protein synthesis inhibition depends on memory reactivation at Re-exposure (PT 1). We performed a similar experiment as in Figure 2, except that mice were not given PT 1 (No Re-exposure). However, mice were micro-infused with ANI or VEH into the dorsal hippocampus at 24 h after training on day 2. Both groups of mice showed comparable memory performance and a progressive loss of escape latencies during training (days 1 and 2) (data not shown). In contrast to the results shown in Figure 2, VEH- and ANI-infused mice searched selectively in the TQ in the probe test (VEH, F_3,32 _= 16.485, P < 0.05; ANI, F_3,32 _= 10.108, P < 0.05) (Figure 3). ANI-infused mice showed comparable searching scores in the TQ as the VEH group (F_1,16 _= 0.002, P > 0.05). These results indicated that the inhibition of protein synthesis failed to disrupt spatial memory when the memory was not reactivated by the Re-exposure session, suggesting that the amnesic effects of ANI are contingent on the reactivation of spatial memory.

### Reconsolidation of spatial memory depends on CREB-mediated transcription

**Figure 4:**
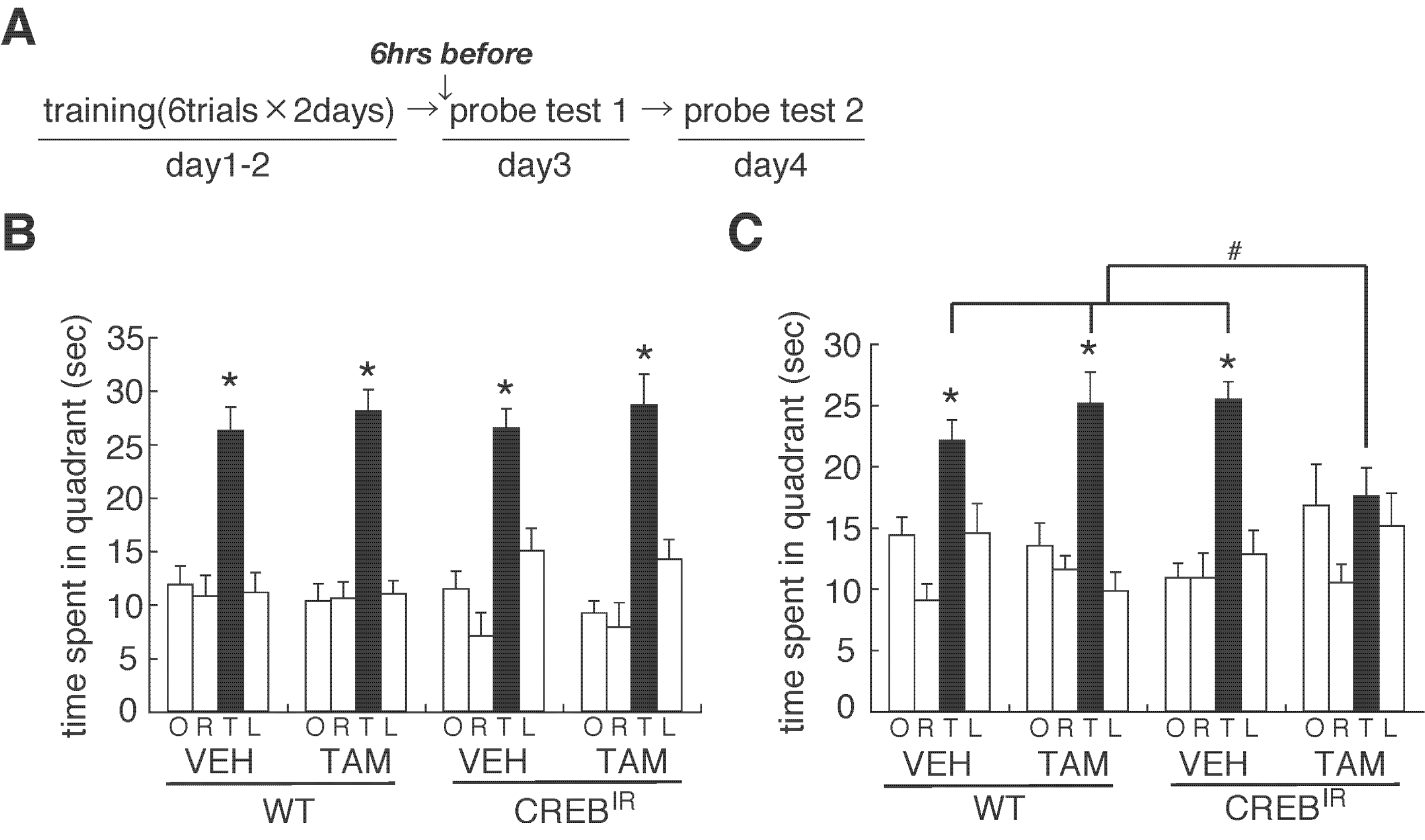
**Role of CREB-mediated transcription in the reconsolidation of spatial memory**. (A) Experimental design. (B) Probe trial at day 3 (PT 1) (WT/VEH, n = 12; WT/TAM, n = 9; CREB^IR^/VEH, n = 9; CREB^IR^/TAM, n = 10). *P < 0.05, time spent in the TQ vs. time spent in the other quadrants by *post hoc *Newman-Keuls test after significant one-way ANOVA. (C) Probe trial at day 4 (PT 2). *P < 0.05, time spent in the TQ vs. time spent in the other quadrants by *post hoc *Newman-Keuls test after significant one-way ANOVA. ^#^P < 0.05, comparisons of searching scores in the TQ among the experimental groups of mice. Error bars are SEM. Time spent (s) in target (T), adjacent left (L), adjacent right (R), and opposite (O) quadrants during the probe trial is shown.

We previously demonstrated that CREB-mediated transcription is required for the reconsolidation of contextual fear memory [13,22,25]. To further understand the mechanism for the reconsolidation of spatial memory, we examined the role of CREB-mediated transcription in the reconsolidation of spatial memory using CREB^IR ^mice [13,22,25] (Figure 4).

We performed a similar experiment as shown in Figure 2, except that CREB^IR ^mice and wild type (WT) littermates received a systemic injection of tamoxifen (TAM; 16 mg/kg, i.p.) or peanut oil (VEH) at 6 h before PT 1 to induce the inhibition of CREB activity (Figure 4A). Similarly with Figure 2, all groups of mice showed comparable memory performance and a progressive loss of escape latencies during training (days 1 and 2) (data not shown). Twenty-four hours after training on day 2, all groups searched selectively and comparably in the TQ during PT 1 (WT/VEH, F_3,44 _= 14.867, P < 0.05; WT/TAM, F_3,32 _= 29.077, P < 0.05; CREB^IR^/VEH, F_3,24 _= 13.937, P < 0.05; CREB^IR^/TAM, F_3,36 _= 22.310, P < 0.05) (Figure 4B). Importantly, our observation that TAM-injected CREB^IR ^mice displayed normal searching scores in the TQ suggests that the genetic inhibition of CREB activity had no effect on the retrieval of spatial memory.

In contrast to these observations, TAM-injected CREB^IR ^mice showed no preference for the TQ in PT 2, while the other groups selectively searched in the TQ (WT/VEH, F_3,44 _= 9.187, P < 0.05; WT/TAM, F_3,32 _= 13.971, P < 0.05; CREB^IR^/VEH, F_3,24 _= 13.516, P < 0.05; CREB^IR^/TAM, F_3,35 _= 1.941, P > 0.05) (Figure 4C). TAM-injected CREB^IR ^mice showed a significant decrease in their searching scores in the TQ compared to PT 1 (F_1,18 _= 10.230, P < 0.05). Consistently, two-way ANOVA comparing searching scores in the TQ in PT 2 revealed a significant drug versus genotype interaction (drug, F_1,33 _= 2.438, P > 0.05; genotype, F_1,33 _= 1.931, P > 0.05; drug vs. genotype, F_1,33 _= 9.389, P < 0.05). TAM-injected CREB^IR ^mice showed significantly lower searching scores in the TQ compared to the other groups (P < 0.05). These observations indicated that the inhibition of CREB-mediated transcription disrupted reactivated spatial memory, suggesting that CREB-mediated transcription is required for the reconsolidation of spatial memory.

### Destabilization of reactivated spatial memory depends on the activation of CB1 and LVGCCs

**Figure 5:**
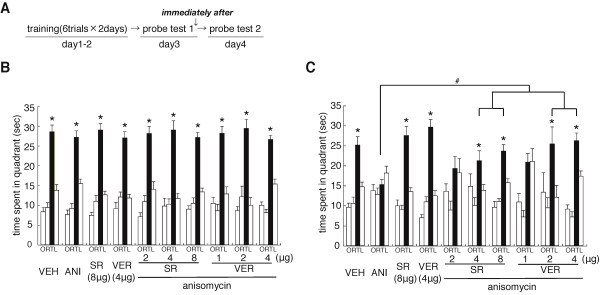
**Roles of CB1 and LVGCCs in the hippocampus in the destabilization of spatial memory**. (A) Experimental design. (B) Probe trial at day 3 (PT 1) (n = 8-15 for all groups). *P < 0.05, time spent in the TQ vs. time spent in the other quadrants by *post hoc *Newman-Keuls test after significant one-way ANOVA. (C) Probe trial at day 4 (PT 2). *P < 0.05, time spent in the TQ vs. time spent in the other quadrants by *post hoc *Newman-Keuls test after significant one-way ANOVA. ^#^P < 0.05, comparisons of searching scores in the TQ among the experimental groups of mice. Error bars are SEM. Time spent (s) in target (T), adjacent left (L), adjacent right (R), and opposite (O) quadrants during the probe trial is shown.

We previously demonstrated that the activation of CB1 and LVGCCs in the hippocampus is required for the destabilization of reactivated contextual fear memory [25]. To understand the mechanisms for the destabilization of reactivated spatial memory, we examined whether the blockade of hippocampal CB1 or LVGCCs would block the amnesic effect of ANI on reactivated spatial memory (Figure 5).

We performed a similar experiment as in Figure 2, except that mice received a micro-infusion of ANI or VEH with either the CB1 blocker SR141716A (SR) or the LVGCC blocker verapamil-HCl (VER) into the dorsal hippocampus immediately after PT 1. All groups of mice showed comparable memory performance and a progressive loss of escape latencies during training (days 1 and 2) (data not shown). Twenty-four hours after training on day 2, all groups of mice searched selectively and comparably in the TQ during PT 1 (VEH, F_3,56 _= 46.208, P < 0.05; ANI, F_3,56 _= 45.649, P < 0.05; SR, F_3,52 _= 60.259, P < 0.05; VER, F_3,48 _= 35.274, P < 0.05; SR 2 μg/μL + ANI, F_3,28 _= 32.731, P < 0.05; SR 4 μg/μL + ANI, F_3,36 _= 26.661, P < 0.05; SR 8 μg/μL + ANI, F_3,52 _= 51.078, P < 0.05; VER 1 μg/μL + ANI, F_3,28 _= 28.652, P < 0.05; VER 2 μg/μL + ANI, F_3,32 _= 21.713, P < 0.05; VER 4 μg/μL + ANI, F_3.56 _= 69.306, P < 0.05) (Figure 5B).

Consistent with the results shown in Figure 2, we observed that the inhibition of protein synthesis disrupted the reactivated spatial memory. However, this disruption by ANI was reversed by the co-infusion of either SR or VER in a dose-dependent manner, while the blockade of CB1 or LVGCCs in the absence of ANI left the spatial memory unaffected (Figure 5C). ANI-, but not VEH-, infused mice failed to selectively search in the TQ during PT 2 (VEH, F_3,56 _= 21.850, P < 0.05; ANI, F_3,56 _= 2.311, P > 0.05). In contrast, mice co-infused with ANI and higher doses of SR (4 or 8 μg/μL) or VER (2 or 4 μg/μL) selectively searched in the TQ (SR, 4 μg/μL, F_3,36 _= 3.992, P < 0.05; 8 μg/μL, F_3,52 _= 25.462, P < 0.05; VER, 2 μg/μL, F_3,32 _= 3.517, P < 0.05; 4 μg/μL, F_3,56 _= 37.015, P < 0.05). Although one-way ANOVA also detected a significant effect of group in the mice co-infused with ANI and low-doses of SR (2 μg/μL) or VER (1 μg/μL) (SR, F_3,28 _= 3.003, P < 0.05; VER, F_3,28 _= 8.882, P < 0.05), these mice failed to display significantly higher searching scores in the TQ compared to the other quadrants. Additionally, SR- or VER-infused mice (without the infusion of ANI) selectively searched in the TQ (SR, F_3,52 _= 31.122, P < 0.05; VER, F_3,48 _= 52.560, P < 0.05).

Two-way ANOVA with drug 1 (VEH vs. ANI) and drug 2 [VEH vs. SR (8 μg/μL) or VER (4 μg/μL)] comparing searching scores in the TQ confirmed the observations described above. We observed significant effects of drug 1 and drug 2, but no significant drug 1 versus drug 2 interaction (SR, drug 1, F_1,54 _= 14.090, P < 0.05; drug 2, F_1,54 _= 8.605, P < 0.05; drug 1 vs. drug 2, F_1,54 _= 2.728, P > 0.05; VER, drug 1, F_1,38 _= 15.151, P < 0.05; drug 2, F_1,38 _= 0.091, P > 0.05; drug 1 vs. drug 2: F_1,38 _= 0.001, P > 0.05). These results reflected the fact that SR- or VER-infused mice showed significantly higher searching scores in the TQ regardless of whether they were co-infused with ANI. The *post hoc *Bonferroni's test showed that ANI-infused mice [without the infusion of SR (8 μg/μL) or VER (4 μg/μL)] displayed a significantly lower searching score in the TQ compared to the other groups (P < 0.05) that displayed comparable searching scores in the TQ (Ps > 0.05). These results indicated that the co-infusion of SR or VER with ANI into the dorsal hippocampus prevented the disruption of reactivated memory by the inhibition of protein synthesis.

A one-way ANOVA comparing searching scores in the TQ among mice co-infused with ANI and with or without low-, middle-, and high-doses of SR (2, 4, and 8 μg/μL) or VER (1, 2, and 4 μg/μL) revealed a significant effect of group (SR, F_3,43 _= 4.235, P < 0.05; VER, F_3,43 _= 5.743, P < 0.05). Mice co-infused with ANI and with middle- or high-doses of SR or VER showed significantly higher searching scores in the TQ than the other groups (P < 0.05). Additionally, mice co-infused with ANI and with low-dose SR or VER showed higher searching scores in the TQ than mice infused only with ANI, although these differences were not statistically significant. These results indicated that SR and VER displayed inhibitory effects on the disruption of reactivated spatial memory by the inhibition of protein synthesis in a dose-dependent manner.

It is important to note that, consistent with previous observations [25], the co-infusion of SR or VER with ANI showed no inhibitory effect on the induction of PTZ-induced c-fos expression by ANI (Additional file 3, Figure S3).

Collectively, these observations indicated that the hippocampal activation of CB1 and LVGCCs is required for the destabilization of reactivated spatial memory.

## Discussion

Previous studies have shown that retrieval temporarily destabilizes memory, and that gene expression-dependent processes are required for subsequent restabilization (memory reconsolidation) [10,12,13,15]. Molecular studies on the regulation of memory stability after retrieval have focused almost exclusively on fear memory, while the underlying molecular mechanisms for the reconsolidation of spatial memory remain poorly understood, except for a few studies showing that the reconsolidation of spatial memory requires mRNA and protein synthesis and the function of protein kinase C (PKC) [14,32-35,40]. In this current study, we first showed that the reactivated spatial memory undergoes reconsolidation, which is dependent on protein synthesis in the hippocampus, confirming previous findings [[33,35], but also [34]]. Using this experimental condition, we investigated the molecular mechanisms by which the reactivated spatial memory is destabilized and restabilized (reconsolidated). We observed that the destabilization of the reactivated spatial memory requires the hippocampal activation of CB1 and LVGCCs, whereas restabilization of the spatial memory requires the hippocampal activation of NMDAR and CREB-mediated transcription.

Previous studies have shown that the activation of NMDAR is required for spatial learning [31,37,41]. Indeed, genetic studies indicated that genetic deletion of the NMDAR-subunits NR1 or NR2A impaired spatial learning [37,41], whereas the forebrain-specific overexpression of NR2B led to the enhancement of spatial learning [42,43]. Consistently, we showed that pharmacological inhibition of NMDAR by AP5 in the dorsal hippocampus impaired spatial learning. Furthermore, we found that this inhibition also blocked the reconsolidation of reactivated spatial memory. Our observations confirmed and extended the previous finding that NMDAR activation is required for spatial learning, and indicated that NMDAR activation is also required for the reconsolidation of spatial memory.

We observed that the post-training, but not pre-training, infusion of AP5 into the dorsal hippocampus failed to impair spatial learning and memory. Previous studies using contextual fear conditioning reported similar observations [44]; a pre-training intra-hippocampal infusion of AP5 impaired memory, while a post-training infusion did not. In contrast to these observations, both pre- and post-infusions of ANI into the dorsal hippocampus blocked the formation of spatial memory. Moreover, mice that received this pre-infusion of ANI showed normal spatial learning during training at day 1, although they displayed impaired spatial memory at the first training session on day 2, indicating that the inhibition of protein synthesis, blocked the formation of spatial memory without affecting spatial learning. Taken together, these observations suggest that the activation of NMDAR and protein synthesis in the hippocampus play distinct roles in spatial learning and memory; the activation of NMDAR is required during the early stages of spatial learning and memory compared to the activation of gene expression that is required in the consolidation phases of memory. However, we could not exclude the possibility that the activation of NMDAR is also required during the initial stage of spatial memory consolidation, as previously reported [45], because the infusion of AP5 after training, which consisted of 6 trials and took longer than 20 min to complete, may be too late to block NMDAR function at the critical time point when NMDAR is required after learning. In addition, in contrast to our observations, previous studies have shown that pharmacological inhibition or hippocampal CA3 region-specific genetic deletion of NMDAR did not affect spatial leaning [46,47]. As discussed above, it is possible that there may be a narrow time window and/or a brain-region specificity when or where, respectively, NMDAR is required for spatial learning [37]. On the other hand, there is a possibility that the pharmacological treatment of ANI or AP5 at day 2 affected reconsolidation of spatial memory that was formed at day 1. However, it is difficult to further examine whether impairments in spatial memory observed in the probe trial at day 3 is due to the inhibition of consolidation or reconsolidation of spatial memory at day 2 because mice could not form a spatial memory at day 1 (6 trials) and required more trainings at day 2 (additional 6 trials) to form a spatial memory in our training protocol (data not shown). Further studies are required to investigate whether contributions of consolidation and reconsolidation to strengthening spatial memory during daily training trials are dissociable.

We previously demonstrated that the reconsolidation of a reactivated contextual fear memory requires the activation of NMDAR [14] and CREB-mediated transcription [13,22,25]. We extended these findings and indicated that the activation of hippocampal NMDAR and CREB-mediated transcription is required for the reconsolidation of reactivated spatial memory. Indeed, pharmacological blockade of hippocampal NMDAR and genetic inhibition of CREB-mediated transcription disrupted reactivated spatial memory. On the other hand, we previously demonstrated that the destabilization of reactivated contextual fear memory requires the hippocampal activation of CB1 and LVGCCs [25]. We also extended these findings and indicated that the activation of CB1 and LVGCCs is required for the destabilization of reactivated spatial memory. Similarly with previous observations, the blockade of CB1 or LVGCCs in the hippocampus by SR or VER, respectively, blocked the disruption of reactivated spatial memory by inhibiting protein synthesis. Thus, reactivated contextual fear and spatial memories undergo destabilization and restabilization (reconsolidation) through similar molecular processes in the hippocampus, raising the possibility that common molecular pathways regulate the stability of these memories in the hippocampus [13,14,20,22,25,33].

Previous study has shown that the pharmacological blockade of NMDAR by pre-, but not post-, re-exposure infusion of a NMDAR blocker into the amygdala impaired the destabilization of reactivated cued fear memory [24]. On the other hand, we observed that this blockade by post-re-exposure infusion into the hippocampus disrupted the reactivated spatial memory, indicating that the blockade of NMDAR in the hippocampus did not affect the destabilization of spatial memory after retrieval. It is possible that the function of NMDAR on destabilization and restabilization differs in brain regions and types of memory. Moreover, there is another possibility that the requirements of NMDAR for destabilization and restabilization of reactivated memory display distinct critical time windows although NMDAR is required for both destabilization and restabilization of reactivated memory.

How do CB1 and LVGCCs cooperatively regulate the destabilization of reactivated memory? LVGCCs are known to be post-synaptically expressed in the excitatory neurons of the hippocampus and increase the post-synaptic Ca^2+ ^concentration in response to depolarization. In contrast, endocannabinoids are released in response to the increase in post-synaptic Ca^2+ ^concentration and then act as a retrograde signal to activate pre-synaptic CB1, leading to decreased neurotransmitter release from the pre-synaptic neuron. Therefore, it is possible that CB1 is activated via the activation of post-synaptic LVGCCs. Furthermore, a recent study has shown that protein degradation via the proteasome is required for the destabilization of reactivated contextual fear memory [23]. Further studies are necessary to investigate the molecular cascades linking CB1, LVGCCs, and proteasome activity to destabilize reactivated memory.

## Methods

### Mice

All experiments were conducted according to the *Guide for the Care and Use of Laboratory Animals, Japan Neuroscience Society *and the *Guide for the Tokyo University of Agriculture*. Male C57BL/6 mice were obtained from Charles River. Transgenic male mice expressing an inducible CREB repressor (CREB^IR ^mice) have been backcrossed to C57BL/6 [13,22,25]. Five or 6 mice were housed in cages, maintained on a 12 h light/dark cycle, and allowed *ad libitum *access to food and water. Mice were at least 8 weeks of age when tested. Testing was performed during the light phase of the cycle. All experiments were conducted blind to the treatment condition of the mouse.

### Surgery

Surgeries were performed as described previously [22,25,38]. Under Nembutal anesthesia and using standard stereotaxic procedures, stainless-steel guide cannulae (22-gauge) were implanted into the dorsal hippocampus (-1.8 mm, ± 1.8 mm, -1.9 mm). Mice were allowed to recover for at least 1 week following surgery. Following this, they were handled for 5 consecutive days prior to the commencement of the Morris water maze.

### Drugs

The protein synthesis inhibitor anisomycin (ANI; 125 μg/μL; Sigma), CB1 antagonist SR141716A (2, 4, or 8 μg/μL) [14,25,48], and LVGCCs antagonist verapamil-HCl (VER; 1, 2, or 4 μg/μL; Sigma) [25] were dissolved in vehicle solution, containing 3 drops of Tween 80 in 2.5 mL of 7.5% dimethyl sulphoxide in artificial cerebrospinal fluid (ACSF) and adjusted to pH 7.4 with NaOH. The NMDA receptor antagonist D-AP5 (1, 2, or 4 μg/μL; Sigma) was dissolved in ACSF. For experiments using the CREB^IR ^mice, 4-hydroxytamoxifen (TAM; 16 mg/kg, i.p.; Sigma) was dissolved in 10 mL of peanut oil (Sigma) [13,22,25].

### Morris water maze

The water maze apparatus and procedures have been described previously [14,28]. Mice were trained with 6 trials per day for 2 days (at 1 min intervals) (training). Spatial learning and memory were assessed in probe trials with the platform removed from the pool. In probe trials, mice were allowed to swim for 60 s and the time spent in each quadrant of the pool was scored.

For the acquisition and formation of spatial memory experiment (Figure 1), mice were infused with vehicle (VEH) or drugs (ANI or AP5) into the dorsal hippocampus at 30 min before or immediately after training. Twenty-four hours following the training session on day 2, mice performed a probe trial. For the reconsolidation experiment (Figure 2), mice were trained and given the first probe trial (PT 1; Re-exposure) as described above. Mice were infused with VEH or drugs (ANI and AP5) into the dorsal hippocampus immediately after PT 1. Twenty-four hours after PT 1, mice performed the second probe trial (PT 2; Test). In the experiment using CREB^IR ^mice (Figure 4), mice were trained and given probe trials as in Figure 2. Mice received a systemic injection of TAM or peanut oil at 6 h before PT 1. For the destabilization experiment (Figure 5), mice were trained and given probe trials as in Figure 2. Mice were infused with VEH or drugs (ANI, SR, or VER) into the dorsal hippocampus immediately after PT 1. Infusions into the dorsal hippocampus (0.5 μL) were made at a rate of 0.25 μL/min for 2 min. The injection cannulae was left in place for 2 min following the infusion.

Cannulae tip placements are shown in the supplementary data, and only mice with cannulae tips within the boundaries of the hippocampus were included in the data analysis (Additional file 4, Figure S4).

### Contextual fear conditioning test

Mice were trained and tested in conditioning chambers (17.5 × 17.5 × 15 cm) that had a stainless-steel grid floor through which footshock could be delivered [22,25]. Training consisted of placing the mice in the chamber and delivering an unsignaled footshock (2 s duration, 0.4 mA) at 148 s later. Mice were returned to their home cages at 30 s after footshock. Twenty-four hours later, mice were placed back in the training context for 5 min during which freezing behavior was assessed (test).

### Immunocytochemistry

To indirectly examine the impact of CB1 and LVGCCs blockers on protein synthesis inhibition by ANI, we examined the induction of the activity-regulated gene c-fos by the chemical convulsant pentylenetetrazol (PTZ; 50 mg/kg, i.p.). Mice were infused with SR or VER into 1 side of the hippocampus and co-infused with ANI and SR or VER into the other side. Thirty minutes following this, mice were treated with PTZ. Thirty minutes later, all mice were perfused transcardially. Brains were subsequently prepared for immunocytochemistry using an anti-c-fos primary rabbit polyclonal antibody (Ab-5, 1:5000; Calbiochem), as previously described [25,38]. Staining was revealed using the streptavidin-biotin peroxidase method (SAB-PO Kit; Nichirei Biosciences, Inc.). Quantification of c-fos-positive cells in sections (100 μm × 100 μm) of the dentate gyrus (DG) was analyzed with Winroof Ver. 5.6 software (Mitani Corp.). The advantage of using PTZ in these experiments is that it induces high levels of c-fos expression, thereby making it easier to detect any reductions in c-fos expression by pretreatment with ANI.

### Data analysis

Data were analyzed with ANOVA. One-way and *post hoc *Newman-Keuls comparisons were used to analyze the differences of the time spent in each quadrant for each group in the Morris water maze test, and the differences in the freezing scores from the contextual fear conditioning test. One- and two-way ANOVA followed by *post hoc *Newman-Keuls and *post hoc *Bonferroni's comparisons, respectively, were used to analyze the effects of drugs and genotype on the time spent in the target quadrant (TQ) in the Morris water maze test. A repeated ANOVA followed by *post hoc *Bonferroni's were used to analyze the effects of drugs on escape latencies. Planned comparisons using Student's *t *test were used to analyze the differences of the number of c-fos positive cells from immunocytochemistry. All values in the text and figure legends are means ± SEM.

## Competing interests

The authors declare that they have no competing interests.

## Authors' contributions

SK is responsible for the hypothesis development and overall design of the research and experiment, and supervised the experimental analyses. SK and RK co-wrote the manuscript. RK and RM performed behavioral analyses. RK perfomed immunocytochemical analyses. All authors read and approved the final manuscript.

## Supplementary Material

Additional file 1 **Figure S1. Effect of protein synthesis inhibition in the hippocampus by ANI on the induction of c-fos by PTZ treatment**. (A) Images of hippocampal c-fos-positive cells in the indicated mice. (B) Quantitative analyses of c-fos expression in the dentate gyrus (VEH, n = 6; ANI, n = 6). Data for c-fos activation in each group were expressed as the percentage of the averaged values in the VEH group. Error bars are SEM. *P < 0.05 compared to the VEH-infused group by Student's *t *test. Click here for file

Additional file 2 **Figure S2. Results of the contextual fear conditioning test**. Error bars are SEM. Click here for file

Additional file 3 **Figure S3. Effects of blocking CB1 and LVGCCs in the hippocampus on the inhibition of the PTZ-induced expression of c-fos by ANI**. (A) Images of hippocampal c-fos-positive cells in the indicated mice. (B) Quantitative analyses of c-fos expression the in dentate gyrus (n = 4 for all groups). Data for c-fos activation in each group were expressed as the percentage of the averaged values in the SR or VER-infused groups. Error bars are SEM. *P < 0.05 compared to the SR or VER-infused group by Student's *t *test. Click here for file

Additional file 4 **Figure S4. Illustrating cannulae tip placements in the hippocampus**. Coronal drawings showing the placement of the cannulae tip in the experiments shown in Figure 1A-C, Figure 1D-F, Figure 2, Figure 3, and Figure 5. Only mice with needle tips within the boundaries of the hippocampus were included in the data analysis. Click here for file
