# Supplementary figures and images for: Molecular mechanisms for the destabilization and restabilization of reactivated spatial memory in the Morris water maze
Source: Mol Brain. 2011 Feb 11;4:9. doi: 10.1186/1756-6606-4-9 (PMC3045328; doi:10.1186/1756-6606-4-9)

**A**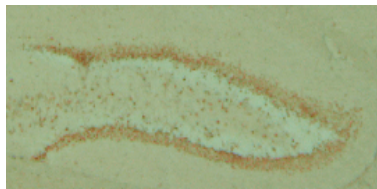**VEH**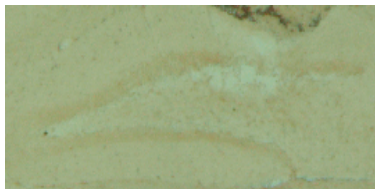**ANI****B**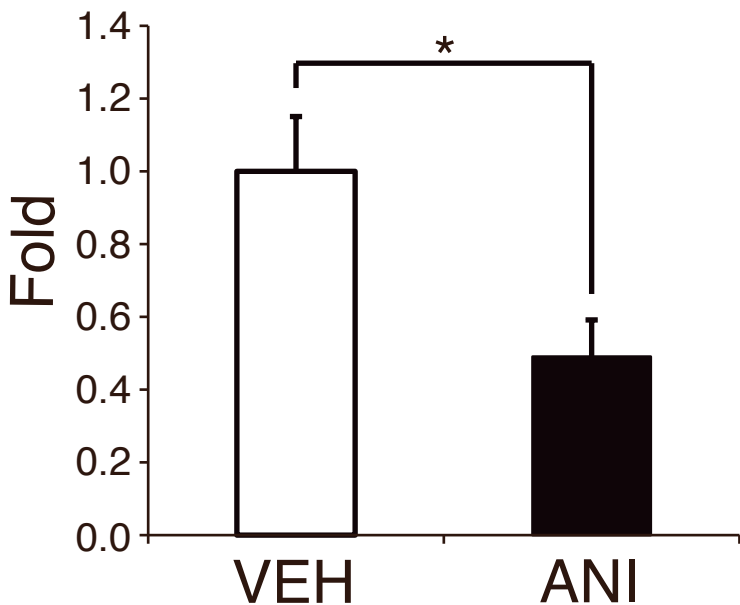

Supplement: Additional file 1 — Figure S1. Effect of protein synthesis inhibition in the hippocampus by ANI on the induction of c-fos by PTZ treatment. (A) Images of hippocampal c-fos-positive cells in the indicated mice. (B) Quantitative analyses of c-fos expression in the dentate gyrus (VEH, n = 6; ANI, n = 6). Data for c-fos activation in each group were expressed as the percentage of the averaged values in the VEH group. Error bars are SEM. *P < 0.05 compared to the VEH-infused group by Student's t test. [file 1756-6606-4-9-S1.PDF]

**A**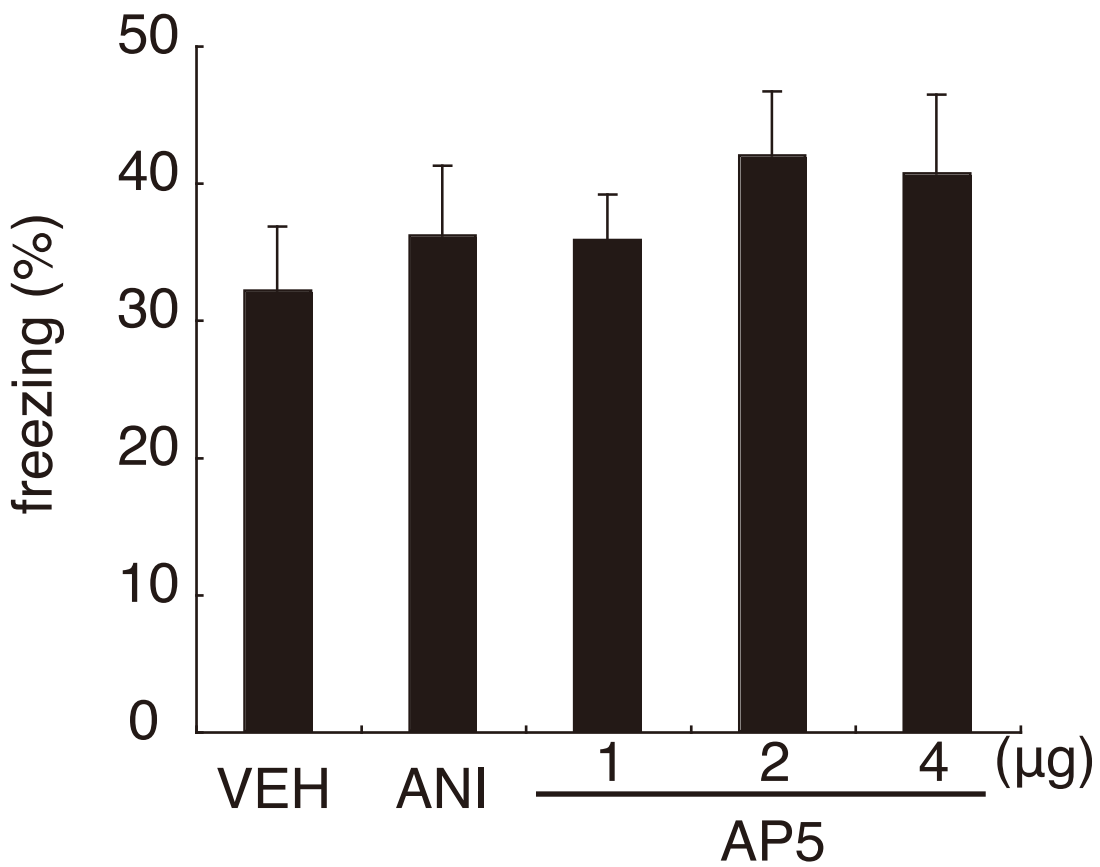**B**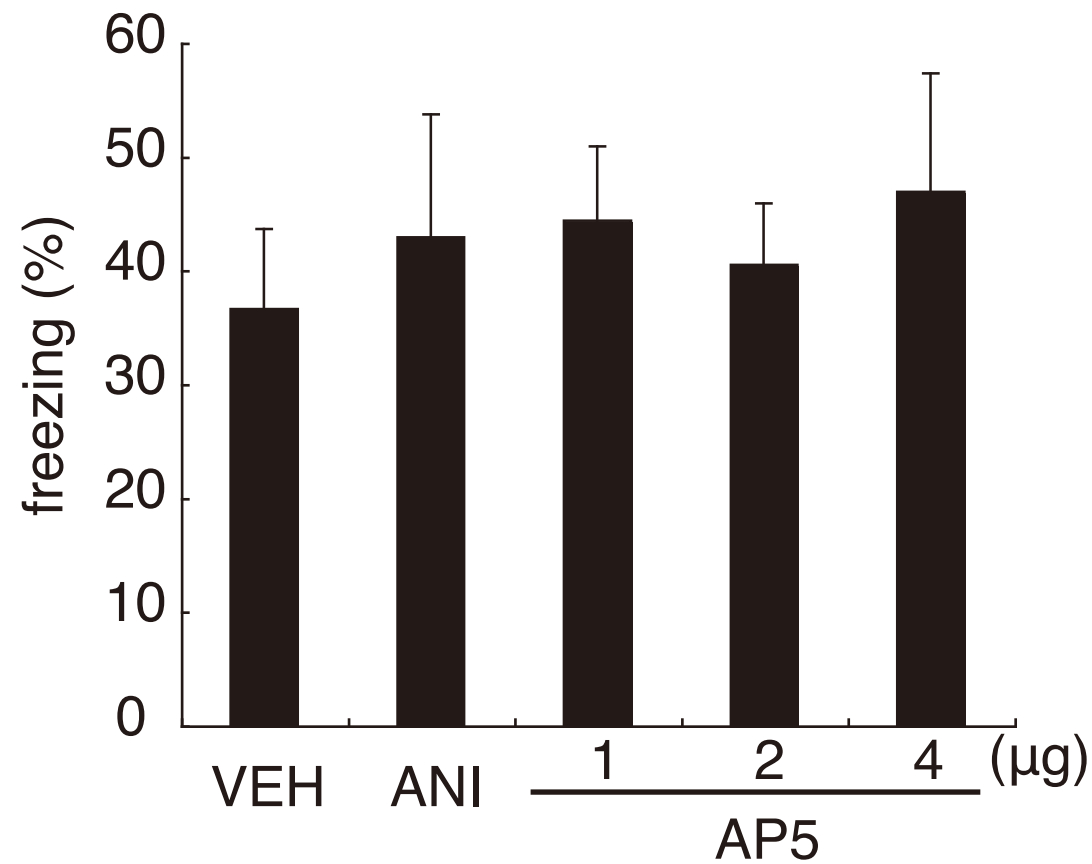

Supplement: Additional file 2 — Figure S2. Results of the contextual fear conditioning test. Error bars are SEM. [file 1756-6606-4-9-S2.PDF]

**A**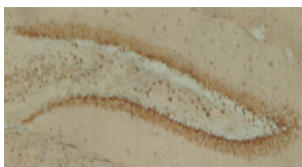**SR**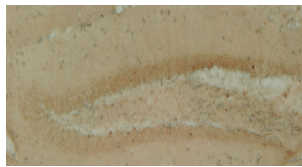**SR/ANI**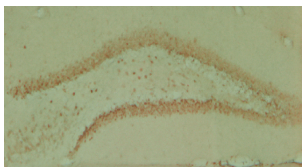**VER**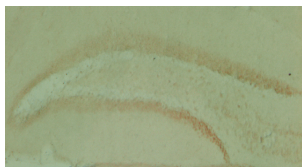**VER/ANI****B**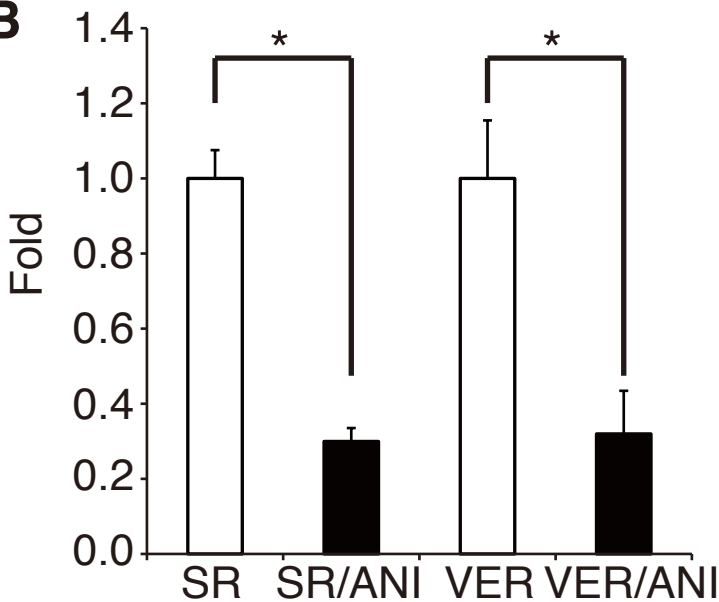

Supplement: Additional file 3 — Figure S3. Effects of blocking CB1 and LVGCCs in the hippocampus on the inhibition of the PTZ-induced expression of c-fos by ANI. (A) Images of hippocampal c-fos-positive cells in the indicated mice. (B) Quantitative analyses of c-fos expression the in dentate gyrus (n = 4 for all groups). Data for c-fos activation in each group were expressed as the percentage of the averaged values in the SR or VER-infused groups. Error bars are SEM. *P < 0.05 compared to the SR or VER-infused group by Student's t test. [file 1756-6606-4-9-S3.PDF]
