# Supplementary material for: Molecular mechanisms for the destabilization and restabilization of reactivated spatial memory in the Morris water maze
Source: Mol Brain. 2011 Feb 11;4:9. doi: 10.1186/1756-6606-4-9 (PMC3045328; doi:10.1186/1756-6606-4-9)
Supplement: Additional file 4 — Figure S4. Illustrating cannulae tip placements in the hippocampus. Coronal drawings showing the placement of the cannulae tip in the experiments shown in Figure 1A-C, Figure 1D-F, Figure 2, Figure 3, and Figure 5. Only mice with needle tips within the boundaries of the hippocampus were included in the data analysis. [file 1756-6606-4-9-S4.PDF]

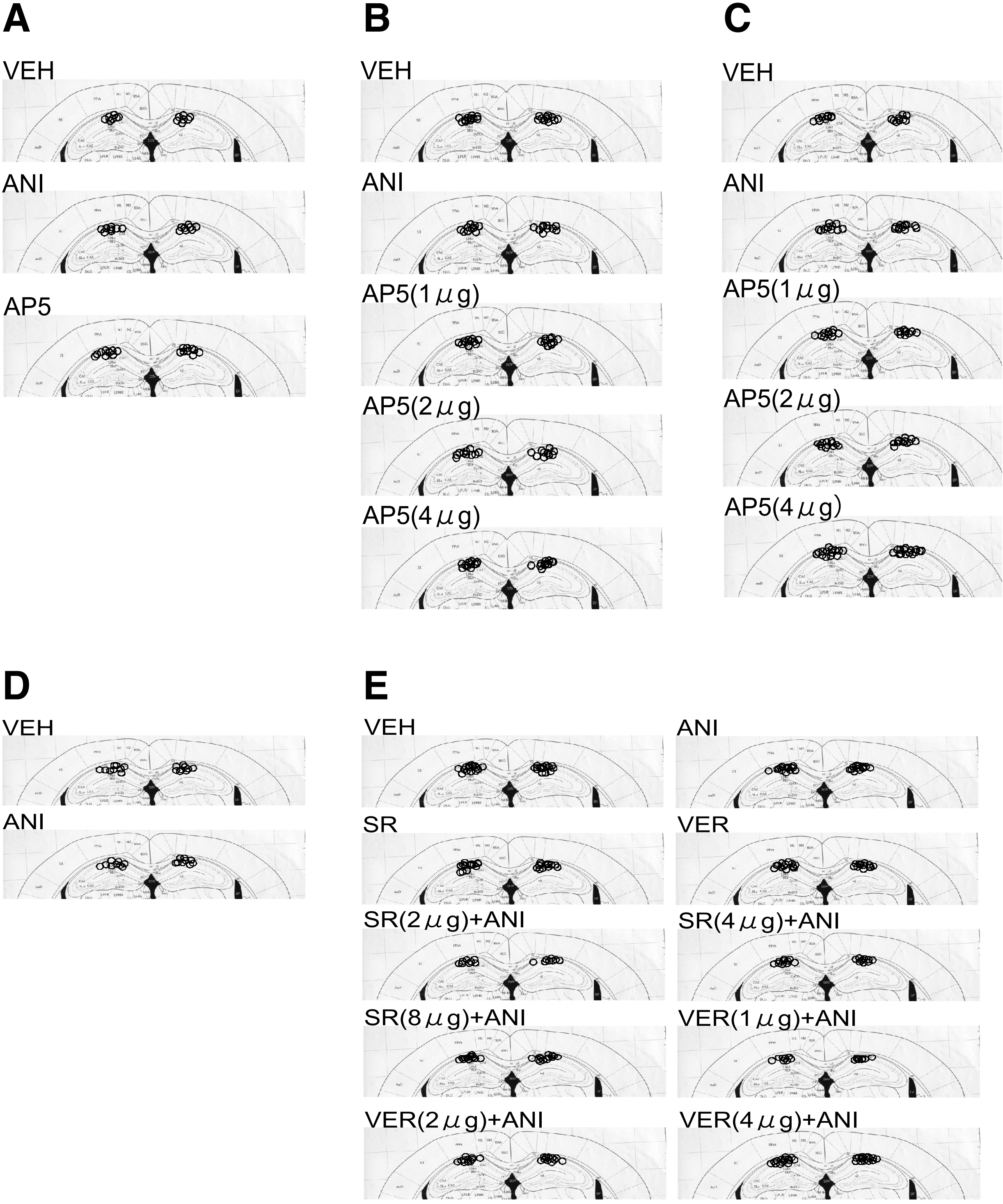
A
VEH
ANI
AP5
B
VEH
ANI
AP5(1μg)
AP5(2μg)
AP5(4μg)
C
VEH
ANI
AP5(1μg)
AP5(2μg)
AP5(4μg)
D
VEH
ANI
E
VEH
ANI
SR
VER
SR(2μg)+ANI
SR(4μg)+ANI
SR(8μg)+ANI
VER(1μg)+ANI
VER(2μg)+ANI
VER(4μg)+ANI
